# Feasibility of rescue stenting technique in patients with acute ischemic stroke due to middle cerebral artery occlusion after failed thrombectomy: A single-center retrospective experience

**DOI:** 10.1371/journal.pone.0274842

**Published:** 2022-09-27

**Authors:** Jang Hun Kim, Jong-Il Choi

**Affiliations:** 1 Department of Neurosurgery, Korea University Anam Hospital, Korea University College of Medicine, Seoul, Korea; 2 Department of Neurosurgery, Korea University Ansan Hospital, Korea University College of Medicine, Gyeonggi-do, Korea; Universitatsklinikum Regensburg, GERMANY

## Abstract

**Background:**

Despite remarkable advancements in intra-arterial mechanical thrombectomy (IAT), recanalization failure rates up to 24% have been reported. Recently, permanent stent placement (rescue stent, RS) during IAT has been suggested as an optional modality for better reperfusion and outcomes in these patients. However, previous studies were limited owing to non-standardized procedure protocols and small sample sizes. Here, we aimed to determine the efficacy and safety of RS in patients with acute ischemic stroke (AIS) with middle cerebral artery (MCA) occlusion.

**Methods:**

Of the 243 patients in our IAT database (2015–2021), 183 were identified as having MCA occlusion alone. Among them, we extracted 53 patients in whom the IAT failed to show thrombolysis in cerebral ischemia (TICI) scores of 2A or worse. Intraoperatively, RS was deployed in 22 patients (RS group), whereas 31 patients (no-stent group) received IAT without stenting. The baseline characteristics and radiologic and clinical outcomes were reviewed. Comparisons between the groups and multivariate logistic analyses for recanalization and good functional outcomes (modified Rankin Scale 0–2) were performed.

**Results:**

No baseline differences were noted (RS *vs*. no-stent); however, the recanalization outcomes (59.1% *vs*. 25.8%, *p =* 0.15) and proportion of good modified Rankin Scale scores (45.5% *vs*. 19.4%, *p =* 0.041) were better in the RS group. The parameters of symptomatic ICH (9.7% *vs*. 9.4%) and mortality (6.5% *vs*. 5.7%) showed no significant difference. In the multivariate analyses, ‘hypertension’ and ‘RS deployment’ were identified as significantly associated factors with recanalization and good prognosis.

**Conclusion:**

In select patients with MCA occlusion AIS after failed IAT, the RS technique can be an optional rescue treatment modality for acquiring better functional outcomes and delayed recanalization.

## Introduction

Intra-arterial mechanical thrombectomy (IAT) is now a standard first-choice therapy for effective recanalization in patients with acute ischemic stroke (AIS) with large vessel occlusion (LVO) within the recently extended time window of 24 h after symptom onset [1, 2]. Compared to medical treatment, IAT is superior with respect to reperfusion of salvageable brain tissue [3–6]. However, despite remarkable advancements in IAT techniques, failure rates of up to 24% have been reported [1, 2]. Recently, permanent placement of a self-expanding stent, the so-called “rescue stent (RS)” technique, has been suggested as an optional modality for failed reperfusion patients and is associated with good outcomes without increasing morbidity or mortality [7–11]. However, previous studies were limited owing to the heterogeneity of the location of the occlusion site, peri-procedural protocols, and small numbers.

We hypothesized that the standardized RS technique would be effective, especially in patients with middle cerebral artery (MCA) occlusion who have undergone failed thrombectomy. It is well understood that underlying ‘atherosclerotic or calcified’ intracranial arterial stenosis (ICAS), which is frequently observed in the MCA, can lead to a higher risk of thrombectomy failure [12]. In this situation, permanent deployment of the stent can be beneficial for widening the arterial diameter; thus, the plasminogen effect can be initiated at thrombosed sites [13]. Consequently, it can salvage a larger volume of the ischemic penumbra in patients prone to ongoing infarction. Herein, we attempted to identify the efficacy of RS by comparing failed thrombectomy patients with deployment of RS to those without.

## Methods

### Patients

**Fig 1:**
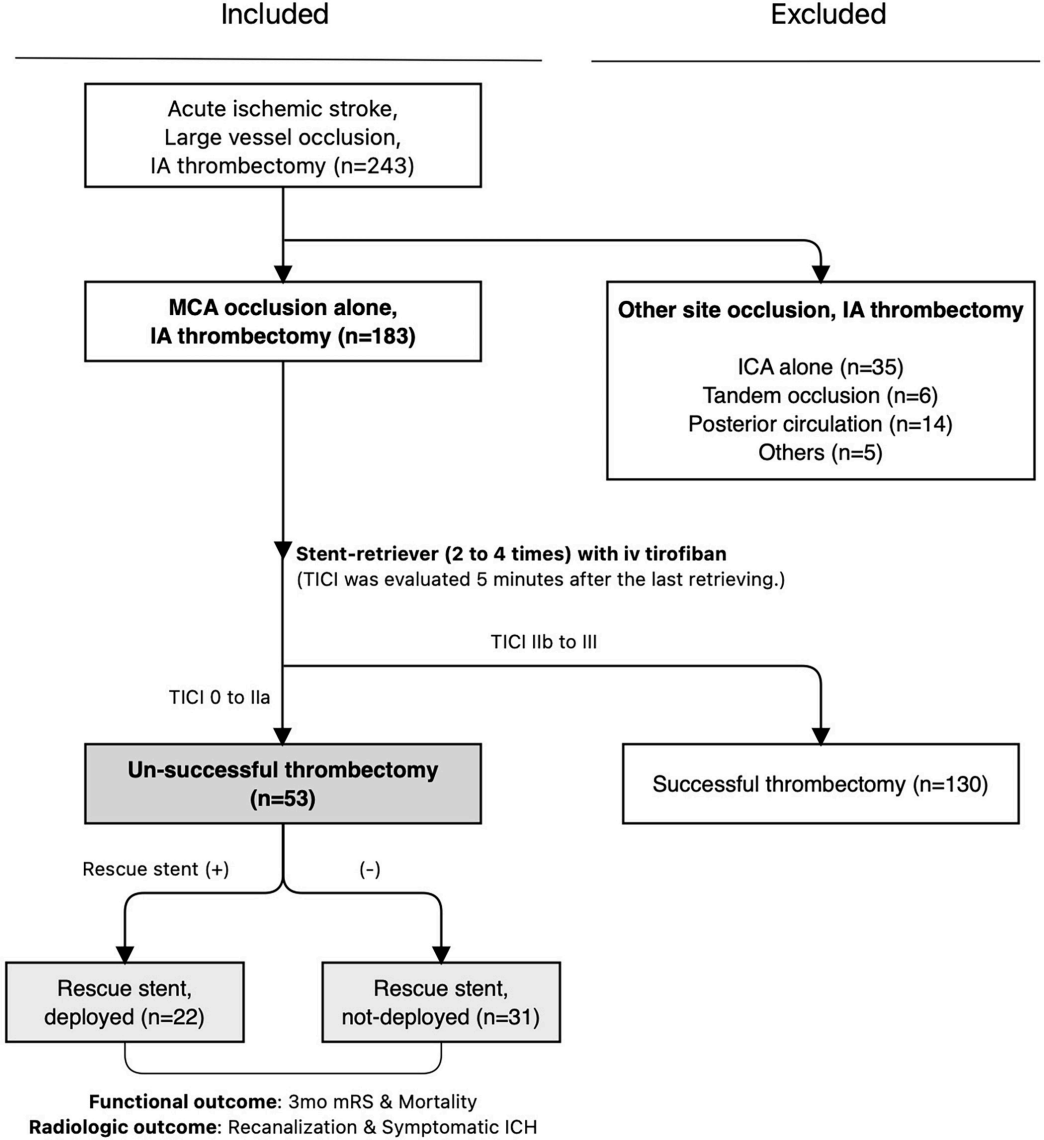
Flowchart of patient enrollment.

Among 243 patients who were diagnosed with AIS due to LVO and who were eligible for emergent IAT from March 2015 to February 2021, we retrospectively extracted 53 patients according to the enrolled criteria: (1) those who were identified as having LVO of the **‘MCA alone’** (2) with confirmed **‘failed’** IAT procedures. ‘Failed thrombectomy’ was defined when thrombolysis in cerebral ischemia (TICI) scores of ‘2A or worse’ were noted at the final angiography after sufficient stentrieving with or without contact aspiration [14]. Patients were excluded if they had other site occlusions (tandem occlusion) or had successful TICI recanalization scores of 2B–3. Tandem occlusion is defined as the lesion involved not only the extracranial (cervical) part of the internal carotid artery (ICA) but also concomitant thromboembolism of its intracranial distal segment or MCA [15]. A flowchart of patient enrolment is shown in Fig 1.

From the case report form (CRF) of our database, we collected data including general information (age, sex, and past medical history), National Institutes of Health Stroke Scale (NIHSS) score at admission, procedural data (use of intravenous tissue plasminogen activator [tPA], onset-to-puncture time, procedure time, and number of retrieval attempts), and clinical course data. Outcomes were evaluated using mortality rates and modified Rankin Scale (mRS) scores 3 months after the intervention.

### Ethics statement

The current study was approved by the Institutional Review Board of the Human Research Center of Korea University Ansan Hospital and the given number of the study is 2022AS0146.

### Informed consent

In every case, informed consent was obtained just before surgery. Patients or legal guardians were informed that the IATs were tailored according to the patient-specific characteristics determined from the clinical and radiological findings and that permanent stents can be deployed with limited evidence. It was obtained by the written forms and the possible side effects and benefits were fully explained.

### Procedures

**Fig 2:**
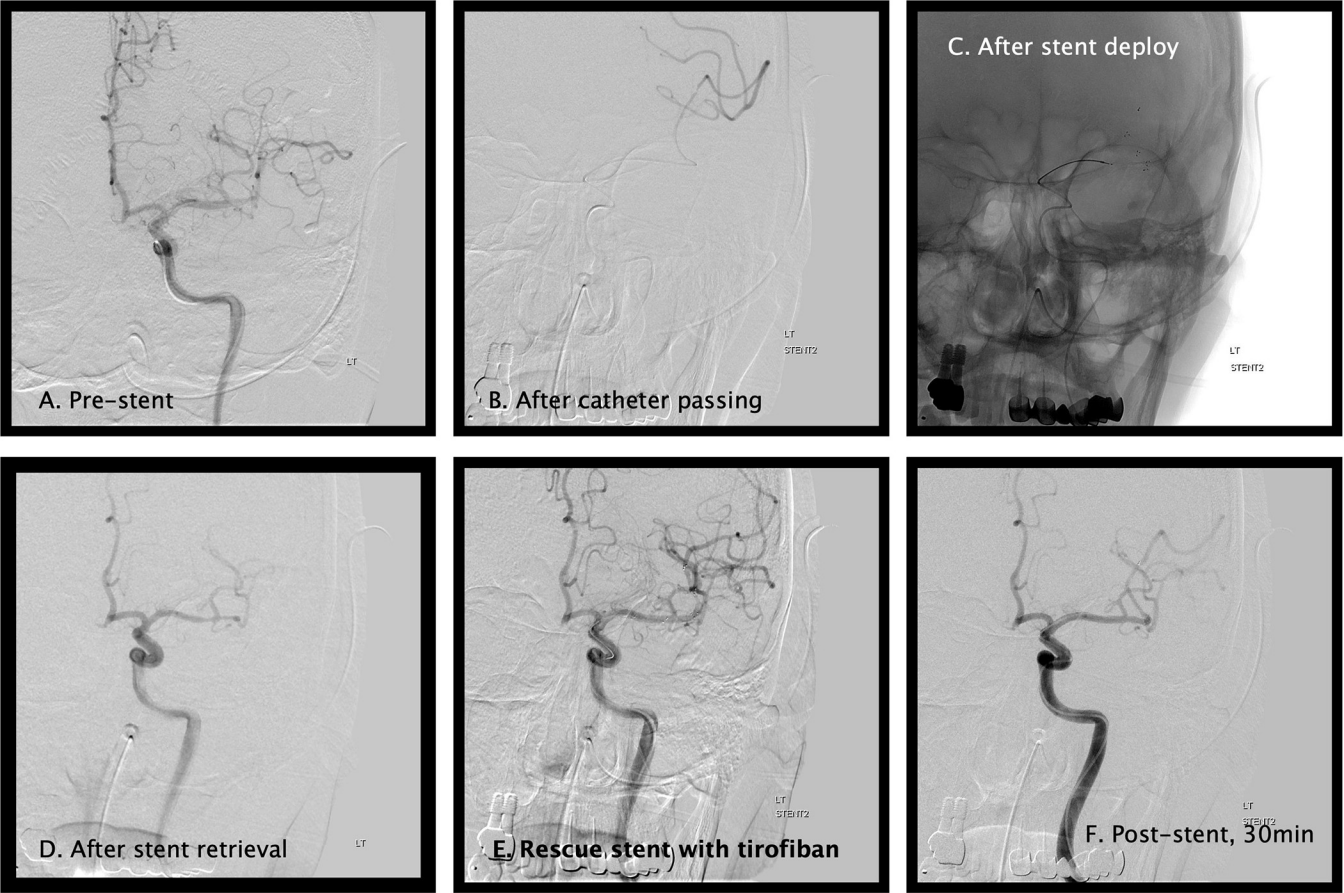
Intraoperative angiograms during RS deployment. The angiogram shows occlusion of the left M2 superior branch (A). The micro-angiogram visualizes the peripheral arterial flows after passing the occlusion site (B). Despite several attempts at stent deploying and retrieving (C), failed thrombectomy of TICI 0 is observed (D). Finally, the RS is permanently deployed (E), and recanalization of the superior M2 is shown after 15 minutes in an angiogram (F). RS, rescue stent; TICI, thrombolysis in cerebral ischemia.

The IAT procedure was performed under local anesthesia with or without mild conscious sedation, according to the patient’s status. The procedure was performed by two independent interventionists. Usually, the target vessel IAT can be directly initiated without performing routine four-vessel angiography, as preoperative computed tomography angiography (CTA) was preoperatively evaluated. A balloon-guiding catheter (8Fr Cello, Covidien/ev3, Irvine, CA, USA) was placed in the relevant cervical ICA, and the intermediate catheter (6Fr Sofia, Microvention, Aliso Viejo, CA) was navigated to the distal ICA or proximal middle cerebral artery (MCA) according to the surgeon’s decision. Contact aspiration thrombectomy was performed after balloon inflation. The procedure was terminated if successful (TICI IIB–III) aspiration was performed. If contact aspiration was unsuccessful, stentriever thrombectomy was followed with **Solitaire FR** (Covidien/ev3, Irvine, CA) or **Trevo Proview** stents (Stryker, Fremont, CA). In this situation, continuous intravenous **tirofiban** (Aggrastat, Medicure Pharma, Princeton, NJ) infusion was administered without exception (loading: 0.4 mcg/kg/min for 30 minutes, maintenance: 0.1 mcg/kg/min for 4~6 hours). At least two to five retrievals were conducted, and angiography was performed to evaluate the TICI score. If thrombectomy was successful (TICI IIB to III), repetitive confirmative angiography was performed 15 min later. When patients presented refractory occlusion after several retrievals (TICI 0 to IIA), the physician decided whether to perform permanent stenting (RS) or stop the operation. Two different protocols were used according to the surgeon’s preference: (1) RS and (2) no-stent. In the RS group, the self-expandable **Solitaire FR** stent was permanently detached in the usual manner, fully covering the expected stenotic or occluded sites of the MCAs (Fig 2). Owing to possible insurance issues, the Wingspan stent (Stryker, Fremont, CA, USA) was not used.

### Radiologic evaluations

**Fig 3:**
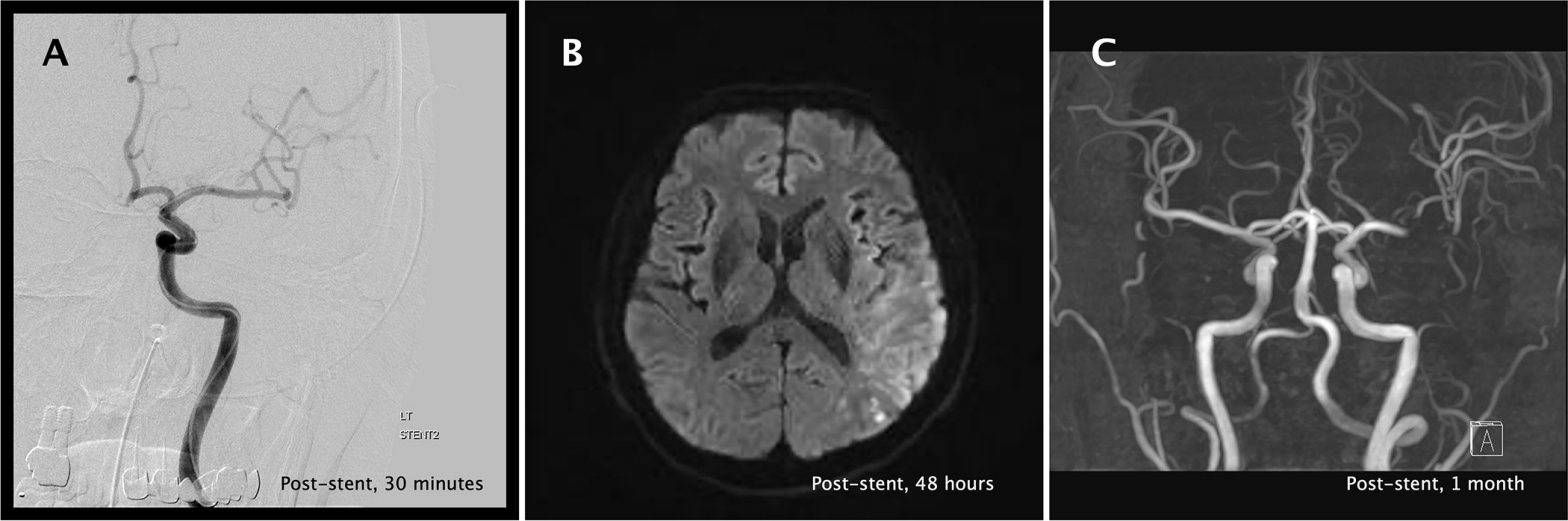
Radiologic images of a patient before and after RS deployment. Before IAT, diffusion-perfusion mismatch was identified by comparing the DWI to CTP images (A). Three months after the procedure, recanalization of M2 was noted on CTA. RS, rescue stent; IAT, intra-arterial thrombectomy; DWI, diffusion-weighted image; CTP, perfusion computed tomography; CTA, computed tomographic angiography.

Before the procedure, head and neck computed tomography (CT), including CTA and perfusion CT (CTP), and magnetic resonance diffusion-weighted imaging (DWI), were performed to identify the infarction core, ischemic penumbra, perfusion-diffusion mismatch (PDM), and origin of the stroke. Perfusion-delayed areas were measured using mean transient time (MTT) CTP sequences, and diffusion restrictions were defined as high signal intensity lesions with b-values of 1000 s/mm^2^ in echo-planar DWI sequences (Fig 3A) using the Alberta Stroke Program Early CT Score (ASPECTS) system [16]. Using the ASPECTS system, [16] PDM was defined as differences of more than 2 points between CTP and DWI [17].

After the IAT procedure, a subsequent CT scan (or DWI) was performed within 48 hours and 7 to 10 days after the onset of stroke or whenever neurological deterioration occurred. A hemorrhage was considered as symptomatic intracranial hemorrhage (sICH) if it was not seen on a previous CT scan and there had subsequently been either a suspicion of hemorrhage or any decline in neurologic status (≥ 4 point increase in the total NIHSS score or an increase ≥ 2 points in one NIHSS category) [18]. Three months after the procedure, angiographic studies (CTA, MRA, or DSA) were performed to evaluate the recanalization. Recanalization was defined as the absence of vessel occlusion and prominent visualization of the distal vessels in the following images (Fig 3B).

### Statistical analysis

Continuous values were presented as means and standard deviations, and categorical variable data were presented as numbers and percentages. A comparison analysis was performed between the two groups (RS *vs*. no-stent groups). In addition, univariate and multivariate logistic regression analyses were conducted to identify the factors associated with good functional outcomes and recanalization. Statistical significance was set at *p* < 0.05. Statistical analyses were performed using standard software (version 23.0, SPSS, IBM, Chicago, IL, USA).

## Results

Among 183 reviewed patients who were diagnosed with LVO-AIS at the MCA, 130 (71%) achieved successful recanalization of TICI 2B to 3. However, 53 enrolled patients (29%) remained non-recanalized after contact aspiration and stentriever thrombectomy (TICI 0 to 2A). The general demographics of the enrolled patients and the results of the comparative analysis between the groups are presented in Table 1. The mean age of the patients was 67 years, and two-thirds of the patients had pathologies of the M1 segments. The initial NIHSS and ASPECTS scores were 14.89 and 7.75, respectively. In terms of the outcomes of the enrolled patients, only 16 patients (30.2%) achieved good mRS scores (0–2) at 3 months, and 3 patients (5.7%) died.

**Table 1:** Results of comparative analysis between the rescue stent and no-stent groups.

Parameters		Enrolled patients (N = 53)		No-stent group (N = 31)		Rescue stent group (N = 22)		Mean differences or odds ratio	95% Confidence interval		*p*-value
**Patients**											
Age		67.62	±5.350	68.32	±5.160	66.64	±5.577	1.686	-1.300	4.672	0.262
Female sex		23	(43.4%)	13	(41.9%)	10	(45.5%)	1.154	0.384	3.471	0.799
HTN		30	(56.6%)	17	(54.8%)	13	(59.1%)	1.190	0.394	3.594	0.758
DM		18	(34.0%)	11	(35.5%)	7	(31.8%)	0.848	0.266	2.707	0.781
DL		18	(34.0%)	9	(29.0%)	9	(40.9%)	1.692	0.536	5.348	0.368
Smoking history		14	(26.4%)	8	(25.8%)	6	(27.3%)	1.078	0.313	3.710	0.905
Previous stroke		6	(11.3%)	4	(12.9%)	2	(9.1%)	0.675	0.112	4.056	0.666
A.fib		15	(28.3%)	9	(29.0%)	6	(27.3%)	0.917	0.271	3.096	0.889
**Clinical and radiologic features**											
Location	M1	35	(66.0%)	20	(64.5%)	15	(68.2%)	0.848	0.266	2.707	0.781
	M2	18	(34.0%)	11	(35.5%)	7	(31.8%)				
	(superior)	13	(24.6%)	8	(25.8%)	5	(22.7%)				
	(inferior)	5	(9.4%)	3	(97%)	2	(9.1%)				
NIHSS		14.89	±2.407	14.77	±2.539	15.05	±2.257	-0.271	-1.629	1.087	0.690
ASPECTS		7.75	±1.108	7.71	±1.243	7.82	±0.907	-0.109	-0.734	0.517	0.729
**Procedures**											
tPA		24	(45.3%)	13	(41.9%)	11	(50.0%)	1.385	0.461	4.155	0.561
Onset-to-puncture time		246.89	±45.428	241.29	±41.532	254.77	±50.344	-13.482	-38.873	11.908	0.291
Procedure time		123.21	±12.329	123.23	±11.295	123.18	±13.934	0.044	-6.924	7.011	0.990
Number of retrieval attempts		2.91	±0.450	2.94	±0.442	2.86	±0.468	0.072	-0.182	0.325	0.572
**Outcomes**											
Recanalization		21	(39.6%)	8	(25.8%)	13	(59.1%)	4.153	1.289	13.384	0.015*
Symptomatic ICH		5	(9.4%)	3	(9.7%)	2	(9.1%)	0.933	0.143	6.110	0.943
Good mRS (0–2) at 3 months		16	(30.2%)	6	(19.4%)	10	(45.5%)	3.472	1.021	11.808	0.041*
Mortality		3	(5.7%)	2	(6.5%)	1	(4.5%)	0.690	0.059	8.125	0.767

In the comparison analysis, baseline characteristics related to patient information, clinical and radiologic features, and procedure-related data showed no significant differences between the groups. However, in terms of outcomes, patients in the RS group showed higher recanalization (59.1% *vs*. 25.8%, *p =* 0.15) and good mRS scores at 3 months (45.5% *vs*. 19.4%, *p =* 0.041) compared to those in the no-stent group. The parameters of symptomatic sICH (9.7% *vs*. 9.4%) and mortality (6.5% *vs*. 5.7%) showed no significant differences.

Table 2 presents the results of the univariate and multivariate logistic regression analyses performed to identify the factors associated with a good mRS score at 3 months. In the present study, hypertension (*p =* 0.007) and RS deployment (*p =* 0.042) were identified as independent prognostic factors for better functional outcomes.

**Table 2:** Results of univariate and multivariate analyses for identifying factors associated with good functional outcomes (3-month mRS 0–2).

Parameters		Poor mRS (n = 37)		Good mRS (n = 16)		Univariate analysis				Multivariate analysis			
						Hazard Ratio	95% CI		*p*-value	Hazard Ratio	95% CI		*p*-value
**Patients**													
Age		68.22	±5.239	66.25	±5.520	0.929	0.824	1.046	0.222				
Female sex		18	(48.6%)	5	(31.3%)	0.480	0.139	1.655	0.245				
HTN		16	(43.2%)	14	(87.5%)	9.187	1.822	46.336	0.007*	10.531	1.932	57.409	0.007*
DM		13	(35.1%)	5	(31.3%)	0.839	0.239	2.941	0.784				
DL		10	(27.0%)	8	(50.0%)	2.700	0.798	9.139	0.110				
Smoking history		7	(18.9%)	7	(43.8%)	3.333	0.922	12.055	0.066*	2.894	.641	13.059	0.167
Previous stroke		4	(10.8%)	2	(12.5%)	1.179	0.193	7.193	0.859				
A.fib		9	(24.3%)	6	(37.5%)	1.867	0.529	6.583	0.332				
**Clinical and radiologic features**													
Location	M1	24	(64.9%)	11	(68.8%)	0.839	0.239	2.941	0.784				
	M2	13	(35.1%)	5	(31.3%)								
NIHSS		15.03	±2.651	14.56	±1.750	0.920	0.716	1.183	0.517				
ASPECTS		7.78	±1.058	7.69	±1.250	0.923	0.539	1.579	0.770				
**Procedures**													
tPA		14	(37.8%)	10	(62.5%)	2.738	0.816	9.189	0.103				
Onset-to-puncture time		247.16	±37.016	246.25	±62.169	1.000	0.987	1.013	0.946				
Procedure time		123.11	±12.982	123.44	±11.063	1.002	0.955	1.051	0.928				
Number of retrieval attempts		2.89	±0.458	2.94	±0.443	1.262	0.332	4.786	0.733				
Rescue stent		12	(32.4%)	10	(62.5%)	3.472	1.021	11.808	0.046*	4.182	1.050	16.656	0.042*

Table 3 presents the results of the logistic regression analyses for recanalization. Similar to previous results, parameters of hypertension (*p =* 0.005) and RS deployment (*p =* 0.016) were identified as significant factors associated with recanalization.

**Table 3:** Results of univariate and multivariate analyses for identifying factors associated with recanalization.

Parameters		No recanalization (n = 32)		Recanalization (n = 21)		Univariate analysis				Multivariate analysis			
						Hazard Ratio	95% CI		P-value	Hazard Ratio	95% CI		P-value
**Patients**													
Age		68.16	±4.900	66.81	±6.005	0.952	0.855	1.060	0.369				
Female sex		14	(43.8%)	9	(42.9%)	0.964	0.317	2.929	0.949				
HTN		13	(40.6%)	17	(81.0%)	6.212	1.697	22.739	0.006*	7.621	1.821	31.887	0.005*
DM		11	(34.4%)	7	(33.3%)	0.955	0.298	3.058	0.938				
DL		9	(28.1%)	9	(42.9%)	1.917	0.602	6.101	0.271				
Smoking history		6	(18.8%)	8	(38.1%)	2.667	0.764	9.312	0.124				
Previous stroke		3	(9.4%)	3	(14.3%)	1.611	0.293	8.863	0.584				
A.fib		8	(25.0%)	7	(33.3%)	1.500	0.447	5.029	0.511				
**Clinical and radiologic features**													
Location	M1	19	(59.4%)	16	(76.2%)	0.457	0.134	1.558	0.211				
	M2	13	(40.6%)	5	(23.8%)								
NIHSS		14.91	±2.347	14.86	±2.555	0.991	0.787	1.249	0.942				
ASPECTS		7.88	±1.008	7.57	±1.248	0.772	0.459	1.298	0.329				
**Procedures**													
tPA		15	(46.9%)	9	(42.9%)	0.850	0.281	2.576	0.774				
Onset-to-puncture time		251.25	±42.635	240.24	±49.711	0.994	0.982	1.007	0.387				
Procedure time		122.97	±12.106	123.57	±12.956	1.004	0.960	1.050	0.861				
Number of retrieval attempts		2.84	±0.448	3.00	±0.447	2.317	0.597	8.986	0.224				
Rescue stent		9	(28.1%)	13	(61.9%)	4.153	1.289	13.384	0.017*	5.245	1.367	20.121	0.016*

## Discussion

The current study demonstrated the efficacy of deploying RS in select patients with LVO-AIS after failed IAT. Patients in the RS group showed significantly better outcomes, good mRS scores at 3 months, and recanalization during follow-up without increased risks of symptomatic ICH or mortality. In addition, hypertension and RS deployment were identified as independent factors associated with recanalization and good mRS scores. This suggests that the RS technique can be a rescue treatment modality for thrombectomy-failed MCA-occlusion in AIS.

Several previous studies have evaluated the efficacy and safety of permanent stenting in LVO [19–21]. However, the efficacy of RS in selective AIS patients with **failed IAT** was recently investigated [9, 10, 22], and most studies reported favorable outcomes in patients with RS. Despite the proven efficacy of recent studies, the RS technique is not the optimal treatment method because of the lack of randomized trials and prospective study designs. The current investigation is a single-center, retrospectively analyzed study that focused on intracranial RS deployment in the MCA. To acquire more evidence of the efficacy of the RS technique, we strictly followed the procedure protocols and standardized every periprocedural setting, except RS deployment, according to the surgeons’ preferences.

Based on the results of recent clinical trials, IAT is now a standard first-line treatment for effective recanalization in select patients with LVO-AIS within the recently extended time window of 24 h after symptom onset [1, 2]. It is clear that IAT is superior with respect to reperfusion of salvageable brain tissue compared to medical therapy alone [3–6]. However, despite remarkable advancements in IAT techniques, a failure rate of up to 24% has been reported in these two trials, and the medical arm of patients (failed IAT patients) showed dismal outcomes [1, 2]. Irrespective of the cause of refractoriness in LVO-AIS, a rescue modality is needed for such refractory cases. In this situation, RS can be ‘easily’ and ‘intraoperatively’ attempted without excessive time consumption or risks [8, 10].

In our study, RS patients presented more favorable functional outcomes (45.5% good mRS scores) than those without stents (19.4%). This may be related to the higher incidence of delayed recanalization of the occluded MCAs (59.1% *vs*. 25.8%). Owing to the self-expanding characteristics of the stent itself, narrow arteries can be widened irrespective of the underlying pathologies [13]. This can lead to fresh blood delivered to the pathologic site with antegrade and retrograde flows. Physiologically, a thrombus or embolic material can be degraded only when plasmin is activated [23]. Delivering blood (or plasminogen) to the pathologic site is essential for recanalization, even if it is a small amount.

We can assume that MCA occlusion is possibly related to embolism or thrombus formation, with or without underlying stenosis. It is often not possible to distinguish between a hard thrombus and intracranial stenosis during the procedure, although there are studies that consider truncal occlusion to have an underlying intracranial stenosis when all branches and bifurcations are clearly visible beyond the occluded segment [22, 24]. However, in the selected patients (failed IAT), we can speculate that underlying intracranial stenosis would exist much more frequently, as it is difficult to pass the occlusion site owing to the underlying calcification or atherosclerotic luminal irregularity on the artery [25]. In this situation, additional permanent stenting would be performed on the underlying stenotic site, which might induce delayed recanalization of the occluded vessel by enlarging the arterial lumen and guiding the inflow of blood.

Stent deployment is sometimes accompanied by the possible side effects of intimal injury, procedure-related thrombosis, and delayed in-stent restenosis. In our protocol, the procedure was strictly performed by covering with intravenous tirofiban. Tirofiban is classified as a platelet aggregation inhibitor that interferes with protein-protein interactions between fibrinogen and platelet integrin receptor GP IIb/IIIa. Recent studies have shown very promising results regarding the safety of intravenous tirofiban during stent deployment, including lowering of the incidence of procedure-related thrombosis without any increase in symptomatic ICHs [26, 27]. In addition, the RS technique requires only one additional catheter passing through the pathologic artery and stent deployment while pulling backward, and the procedural yield is suspected to be much higher considering the small side effects. We can conclude that procedure-related complications can be effectively prevented by administering intravenous tirofiban during the procedure.

Interestingly, a previous history of hypertension was additionally identified as an indicator of recanalization and good mRS scores in multivariate analyses. In acute stroke management, blood pressure should be lowered to maintain blood flow to the ischemic penumbra [28]. After deploying RSs in patients, avoiding low blood pressure is important for reducing in-stent thrombosis and inducing delayed recanalization. However, in the literature, the association between hypertension and functional outcomes or recanalization is controversial [29, 30]. Future investigations with a larger cohort are warranted to identify the mechanism of hypertension in patients with LVA-AIS with or without RS.

The current study has several limitations. First, our results cannot be generalized because of the small number of retrospectively enrolled patients and the lack of a multi-centered design involving the participation of multiple physicians. A prospectively designed randomized trial with a larger cohort is necessary to develop evidence for RS as an optimal treatment. Second, only 3-month mRS scores were reviewed, and the long-term efficacy of RS was not evaluated. Since intracranial stent insertion can induce delayed in-stent restenosis, patients should be followed up. Third, only the Solitaire stent was used because of insurance issues. If more evidence is accumulated, an appropriate stent can be chosen.

## Conclusion

In select patients with MCA-occlusion AIS after failed IAT, the RS technique can be an optional rescue treatment modality for acquiring better functional outcomes and delayed recanalization.

## Supporting information

S1 Data (XLSX)
